# Comparative Physicochemical Characterization of Maltodextrins Derived from Starches of Red-, Purple-, and Light-Fleshed Potato Cultivars (*Solanum tuberosum* L.)

**DOI:** 10.3390/molecules31122121

**Published:** 2026-06-16

**Authors:** Dorota Gumul, Justyna Rosicka-Kaczmarek, Magdalena Orczykowska, Marcin Łukasiewicz, Karolina Miśkiewicz, Joanna Sobolewska-Zielińska, Anna Areczuk

**Affiliations:** 1 Department of Carbohydrate Technology and Cereal Processing, Faculty of Food Technology, University of Agriculture in Krakow, 122 Balicka St., 30-149 Krakow, Poland; anna.areczuk@urk.edu.pl; 2 Institute of Food Technology and Analysis, Faculty of Biotechnology and Food Sciences, Lodz University of Technology, 2/22 Stefanowskiego Street, 90-537 Lodz, Poland; justyna.rosicka-kaczmarek@p.lodz.pl (J.R.-K.); karolina.miskiewicz@p.lodz.pl (K.M.); 3 Department of Occupational Safety Engineering, Faculty of Process and Environmental Engineering, Lodz University of Technology, Stefanowskiego 2 St., 90-432 Lodz, Poland; magdalena.orczykowska@p.lodz.pl; 4 Department of Engineering and Machinery for Food Industry, Faculty of Food Technology, University of Agriculture in Krakow, 122 Balicka St., 30-149 Krakow, Poland; marcin.lukasiewicz@urk.edu.pl; 5 Department of Food Analysis and Quality Assessment, Faculty of Food Technology, University of Agriculture in Krakow, Al. Mickiewicza 21, 31-120 Krakow, Poland; joanna.sobolewska-zielinska@urk.edu.pl

**Keywords:** purple/red/yellow potatoes, maltodextrin, physicochemical properties, rheological properties

## Abstract

The objective of this study was to examine the physicochemical properties of maltodextrins derived from starch isolated from red- and purple-fleshed potatoes, in comparison to those obtained from light-fleshed potatoes. The investigation focused on several parameters, including dextrose equivalent (DE), non-carbohydrate components, maltooligosaccharide profile, particle size, surface morphology, water-binding capacity, solubility, rheological properties, structural composition as determined by Fourier transform infrared spectroscopy (FT-IR), and molecular weights. Maltodextrins sourced from the starch of colored potato varieties exhibit superior functional properties, notably nearly 100% solubility and enhanced water absorption capacity. This is attributed to their fine microstructure, which promotes hydration and facilitates the diffusion of water into the interior of the particles, in contrast to maltodextrins derived from the starch of yellow potato varieties. This phenomenon is also influenced by the maltooligosaccharide profile, characterized by a high proportion of low-molecular-weight sugars, lower molecular weights, and polydispersity (Pd), as well as the low SPAN of these maltodextrins. Additionally, maltodextrins derived from the starch of yellow potato varieties (Tajfun and Lord) formed soft gels, whereas those from colored potatoes resulted in hard gels.

## 1. Introduction

Maltodextrins are derived from the partial enzymatic hydrolysis of starch [1,2,3,4]. As hydrolysis products, they comprise a mixture of saccharides, including D-glucose and maltose, as well as various oligosaccharides and polysaccharides, such as maltotriose and combinations of maltotetraose and maltopentaose, resulting in a broad molecular weight distribution [1,2,5,6]. It is well established that maltodextrins are classified based on the dextrose equivalent (DE) value, which can reach up to 20. The DE value quantifies the number of aldehyde groups at the reducing ends relative to pure glucose at the same concentration. Consequently, a high DE indicates extensive hydrolytic conversion and a lower average molecular weight compared to maltodextrins with a low DE [2]. Therefore, DE is inversely related to the number-average molecular weight (Mn) and degree of polymerization (DP), which are commonly employed to describe the size distribution of polysaccharide chains in a carbohydrate polymer. The significance of Mn pertains to the prediction of colligative properties, such as boiling and freezing points, whereas DP relates to the mobility of the polymer chain, with longer chains exhibiting restricted movement [7]. It is assumed that the higher the DE value of maltodextrin, the lower the degree of linearity, the lower the average molecular weight, the simpler the molecular structure, the lower the degree of aging, the solubility, sweetness, hygroscopicity, permeability, fermentation, the greater the browning reaction and the freezing point drop, the worse the organization, viscosity, stability and resistance to crystallization. Additionally, the solubility and permeability of maltodextrin increase while the viscosity, cohesion, and resistance to crystallization decrease when the DE value increases [8,9,10]. It should be emphasized that a very important application property of maltodextrins is their very high water-binding capacity and 100% solubility, unlike native starch from which maltodextrins are derived; this is a consequence of high DP and low molecular weight, resulting in the open microstructure of maltodextrins [10].

It is important to note that the physicochemical and functional properties of maltodextrins are influenced by a variety of factors [4,11,12]. Maltodextrins with similar DE values may display different physicochemical properties, as they can be produced from starches of diverse botanical origins, which vary in structure and in the amylose-to-amylopectin ratio. Additionally, variations in the parameters of enzymatic starch degradation may occur. Thus, it is feasible to produce maltodextrins with similar DE values but differing proportions of high- and low-molecular-weight saccharides by modifying the hydrolysis conditions. These variations result in maltodextrins with distinct physicochemical properties. Specifically, stability and solubility are influenced by high-molecular-weight components, whereas viscosity, crystallinity, and sweetness depend on the predominance of low-molecular-weight components [2,11,13,14]. Furthermore, it can be said that the physicochemical and functional properties of maltodextrins are also influenced by the type of enzyme [15] and the type of chemical modification of the starch used as the basis for their production [1]. Research shows that the enzymatic hydrolysis of chemically modified starches (phosphorylation) results in maltodextrins with different physicochemical and functional properties compared to maltodextrins derived from natural starch. Such compounds can be used as stabilizers in two-phase systems, such as emulsions, suspensions, or foams [1]. Kędziora et al. [16] studied the effect of the type of chemical modification of starch—and thus the presence of additional chemical groups and bonds—on the rheological and functional properties of maltodextrins. They found that the products of enzymatic hydrolysis of oxidized starch and starch octenyl succinate exhibited higher surface activity compared to their starch counterparts. Konował et al. [17] also described the effect of chemical modification of starch on its surface properties, noting that hydrolyzates of acetylated starch were capable of reducing the surface tension in a water–air system, with this effect depending on the degree of starch substitution.

Understanding the physicochemical and functional properties of maltodextrins is crucial, given their extensive application in food products. The prevalent use of maltodextrins in the industry is attributed to their safety for food use, as evidenced by their GRAS status, similar to the starch from which they are derived (FDA, 21CFR 184). Maltodextrins are incorporated into food products due to their bulking, textural, emulsifying, and stabilizing properties, as well as their ability to mitigate a starchy aftertaste. Additionally, they serve as sweetness modifiers, agents for controlling non-enzymatic browning, and are used to lower the setting temperature in mixtures. They also function as carriers for bioactive compounds and as materials for the walls of capsules containing active substances or flavors [7,11,12,13,18,19,20,21]. Maltodextrin is a pivotal component in the production of dehydrated foods, as it reduces viscosity and enhances the firmness of these products. These properties of maltodextrins are derived from their capacity to absorb water, form protective barriers on active absorbing surfaces, and increase the glass transition temperature [22].

Starch derived from potatoes with colored flesh has exhibited notable physicochemical and functional properties in comparison to starch typically sourced from potatoes with white or yellow flesh [23,24]. Consequently, it is plausible to hypothesize that the properties of maltodextrins obtained from these two starch types may also differ. Therefore, it is pertinent to examine and contrast the properties of maltodextrins derived from red and purple-fleshed potatoes with those of commonly utilized maltodextrins from light-fleshed potato starch. This is particularly relevant given that red and purple-fleshed potatoes exhibit greater disease resistance and could serve as alternative sources of starch and its hydrolysis products, such as maltodextrins. Considering the botanical origin of starch and its impact on the physicochemical and functional properties of maltodextrins, alongside the extensive application of maltodextrins in food technology, this study aimed to investigate the physicochemical properties of maltodextrins obtained from starch isolated from red- and purple-fleshed potatoes, in comparison with those from light-fleshed potatoes. The study focused on parameters such as DE, non-carbohydrate components, maltooligosaccharide profile, particle size, surface morphology, water-binding capacity and solubility, rheological properties, structural composition (FT-IR), and molecular weights of the starch hydrolysates.

## 2. Results and Discussion

### 2.1. Dextrose Equivalent (DE) of Maltodextrins

Table 1 presents the dextrose equivalent (DE) of maltodextrins obtained from starches isolated from yellow-fleshed potato varieties Tajfun (MT) and Lord (ML), purple-fleshed potato varieties Blue Star (MBS) and Violetta (MV), and the red-fleshed potato variety Magenta Love (MML).

The determined dextrose equivalents (DE) of the maltodextrins examined exhibited minimal variation. The lowest DE value, 15%, was observed in maltodextrin derived from the red-fleshed Magenta Love potato variety. Conversely, the highest DE value, 18%, was identified in maltodextrins sourced from the Tajfun (yellow flesh) and Violetta (purple flesh) varieties. Notably, maltodextrin obtained from the starch of Lord variety potatoes with yellow flesh exhibited a DE value that was 2 units lower than that of maltodextrin from the starch of Tajfun variety potatoes with the same flesh color. In contrast, maltodextrin from the starch of Blue Star potatoes (purple flesh) had a DE value 1 unit lower than that of maltodextrin from the starch of Violetta potatoes (Table 1). The functional properties of maltodextrins are correlated with the DE value and empirically serve as a guide for determining their applications [2]. It is important to note that the dextrose equivalent (DE) does not adequately characterize the oligosaccharide spectrum of hydrolysates, such as maltodextrins. Variations in the quality of the starting material, specifically starch, including the degree of branching, amylose content, amylose/amylopectin ratio (AM/AP ratio), and molecular weights, significantly influence the oligosaccharide composition of the hydrolysates produced through the action of alpha-amylase during maltodextrin production [2,11,13].

### 2.2. Particle Size Distribution of Maltodextrins

Upon examining the particle size distribution of various maltodextrins, it was observed that those derived from potatoes with purple flesh (Violetta variety) and red flesh (Magenta Love variety) exhibited the highest proportion of small particles, specifically those under 30 micrometres. Conversely, the smallest proportion of particles of this size was identified in maltodextrin from potatoes with light-colored flesh of the Tajfun variety (Table 2). The proportion of particles within the 30–70 µm range was most prominent in maltodextrin derived from the starch of light-fleshed Tajfun potatoes, followed by starch from the purple-fleshed Blue Star variety, and subsequently the Lord, Violetta, and Magenta Love varieties (Table 2). In contrast, particles exceeding 70 µm constituted approximately 0.76% solely in maltodextrin from the starch of light-fleshed Tajfun potatoes. Overall, it can be inferred that maltodextrins from the starch of light-fleshed potato varieties (Tajfun and Lord) were characterized by smaller particle sizes compared to those obtained from the starch of red-fleshed Magenta Love and purple-fleshed Violetta potatoes, with the exception of Blue Star maltodextrin, which contained nearly 81% of particles under 30 micrometres (Table 2). The SPAN coefficient, a dimensionless parameter used to evaluate the uniformity of particle size distribution, was also calculated. This coefficient, also referred to as the spread or polydispersity coefficient, describes the breadth of the particle size distribution within a sample. It was determined based on the results of particle size analysis using laser diffraction measurements. A significant finding was that the SPAN coefficient in the analyzed maltodextrins ranged from 2 to 3, indicating that all samples exhibited high heterogeneity, characterized by the presence of particles of varying sizes, with the maltodextrin sample from the yellow-fleshed Tajfun potato variety performing the worst. Maltodextrins derived from the starch of potatoes with colored flesh exhibited the lowest SPAN values, as the SPAN for all of them is 2.0 (Table 2).

### 2.3. Scanning Electron Microscopy of Investigated Maltodextrins

**Figure 1:**
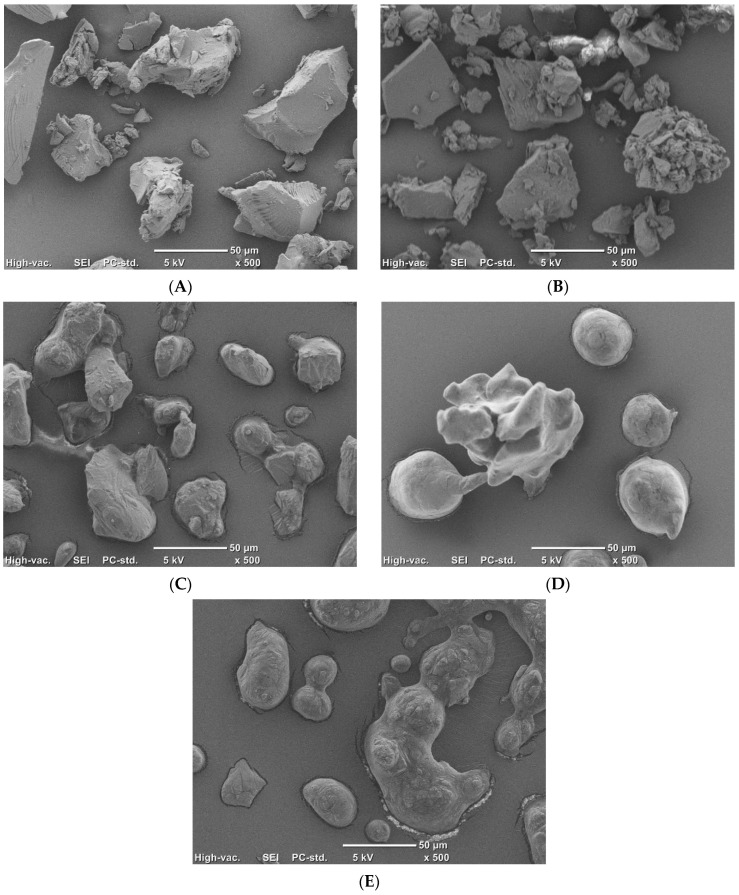
SEM of maltodextrins prepared from starch isolated from yellow potato varieties ((**A**) MT, (**B**) ML) and colored potato varieties ((**C**) MBS, (**D**) MV; (**E**) MML).

The scanning electron microscopy (SEM) images of the maltodextrin samples revealed that the laboratory-produced maltodextrins lacked the granular structure characteristic of native starches. Instead, they exhibited jagged, uneven fragments with non-standard, irregular shapes, often accompanied by cracked walls (Figure 1A–E). This observation aligns with the fact that the enzyme acted on liquefied (gelatinized) starch rather than on intact grains of native starch. The variations among the individual preparations were minimal, suggesting that the hydrolysis and drying processes exert a more significant influence on the final morphology of maltodextrins than the potato variety from which the starch was derived. However, in the case of starch with a higher gelatinization temperature, individual grain elements may be present, as seen in instances of incomplete gelatinization [2,25].

In the maltodextrin group derived from potato varieties with colored flesh, the particle surface generally appeared more fractured and irregular, while a finer fraction was observed in the particle size distribution analysis, with the proportion of particles < 30 µm exceeding 95% for Violetta and Magenta Love (Table 2, Figure 1D,E). In SEM, this is reflected in a greater number of fine agglomerates and fragments with distinct edges and microporosity, which facilitate rapid ‘wetting’ and reconstruction in water. The structure of maltodextrins is associated with high solubility, reaching nearly 100%, and increased water absorption capacity, as demonstrated in Section 2.7. This may be attributed to their fine microstructure, which enhances hydration and facilitates water diffusion into the interior of the particles [2].

The microstructure of maltodextrins derived from the yellow Lord potato variety was found to be intermediate (Figure 1B), characterized by fragments with blurred edges that are less porous than those from colored varieties, yet relatively fine. This structure is consistent with favorable solubility and water-binding capacity (WBC). In contrast, the Tajfun variety exhibited a more diverse range of particle sizes in the SEM observations (Figure 1A), ranging from very fine to larger, compact elements. This corresponds to a broader particle size distribution and the highest SPAN (2.86), indicating high sample heterogeneity (Table 2). Such microstructural heterogeneity may result in uneven hydration, as reflected in the solubility and water absorption results presented in Section 2.7, where this preparation exhibited the lowest solubility and WBC values among all tested samples.

The observed irregular and fragmented shapes are a direct result of the hydrolysis of the starch paste, followed by drying and grinding. In systems with higher molecular weight and extremely broad polydispersity, such as the MT sample with very high Mw and Pd, aggregates and compact fragments appear more frequently. Conversely, systems rich in low-DP fragments, such as the MV and MML samples, form finer, more ‘open’ structures that facilitate wetting and dissolution. These observations align with the understanding that maltodextrins lack a granular structure, with very few granular fragments present in systems with incomplete gelation. The SEM images support the relationship that a higher degree of depolymerization (DP) and a lower molecular weight distribution (MWD) result in a finer and more open microstructure of the maltodextrins, ensuring better hydration and solubility in water (WBC) [26,27]. The opposite is true for maltodextrins with high molecular weights derived from the yellow potato variety (Tajfun).

### 2.4. FT-IR Analysis of Maltodextrins

**Figure 2:**
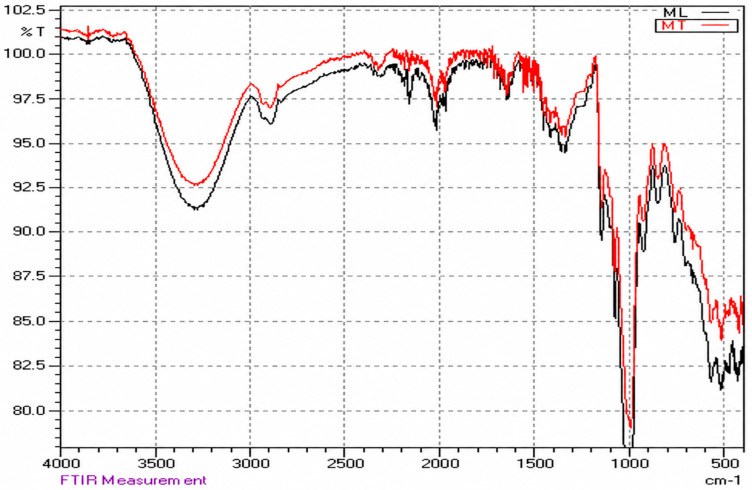
FTIR spectra of maltodextrins derived from starch isolated from yellow potato varieties (Lord, Tajfun) in the 4000–600 cm^−1^ range.

**Figure 3:**
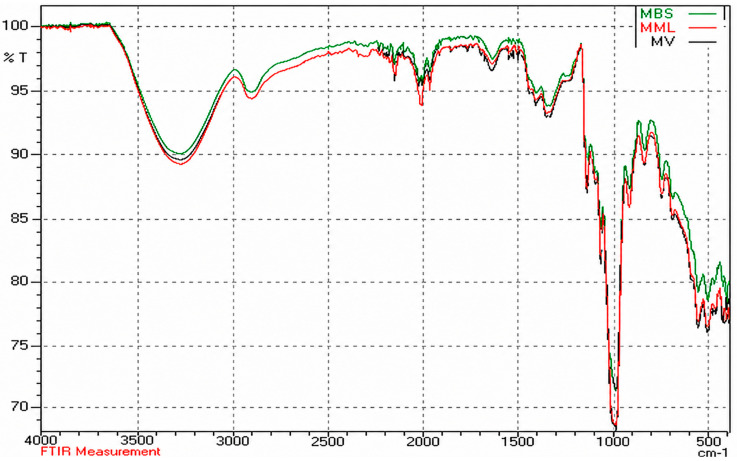
FTIR spectra of maltodextrins derived from starch isolated from colored potato varieties (Violetta, Blue Star, Magenta Love) in the 4000–600 cm^−1^ range.

The FTIR differences between maltodextrins derived from the starch of colored and yellow potato varieties are shown in Figure 2 and Figure 3.

The FTIR spectra obtained for all the maltodextrins examined exhibit characteristic bands typical of polysaccharides: a broad ν(O–H) band at 3600–3000 cm^−1^, indicative of the hydrogen bonding network, with the band and its width being sensitive to hydration; ν(C–H) at 2925–2850 cm^−1^, corresponding to aliphatic chains; δ(H–O–H) at 1645 cm^−1^, representing bound water; and the diagnostic region at 1200–900 cm^−1^, associated with C–O, C–C, and C–O–C in the glucopyranose backbone. Within the ‘fingerprint’ region, a triplet is discernible: ~1047 cm^−1^, associated with local order and referred to as the ‘crystalline’ component; 1022 cm^−1^, representing the amorphous component; and 995 cm^−1^, a band highly dependent on matrix hydration and the local hydrogen-bond network (Figure 2 and Figure 3). A similar tendency was described in the studies on FTIR spectroscopic evaluation of sucrose-maltodextrin-sodium citrate bioglass by Sritham and Gunasekaran [28].

The A_1047_/A_1022_ ratio is frequently employed to assess the short-range order in starch-based materials [29,30].

In maltodextrins derived from starch isolated from colored potato varieties (Violetta, Blue Star, and Magenta Love), an elevated ratio of the 1047 cm^−1^ signal to the 1022 cm^−1^ signal (i.e., a higher A_1047_/A_1022_) was observed (Figure 3), indicating increased structural order and a more coherent hydrogen-bond network. This observation is consistent with the degree of polymerization (DP) profiles obtained for these maltodextrins, discussed in Section 2.5, which showed a higher proportion of DP1–DP6. It is also supported by the molecular weight distribution (MWD) parameters discussed in Section 2.6, which were characterized by lower Mn and Mw values and a low polydispersity index (Pd) of 1.3–1.6. In terms of functional properties, this correlates with nearly 100% solubility and an enhanced water-binding capacity (WBC) (Section 2.7). Conversely, maltodextrin from the Tajfun potato variety exhibits signal enhancement at 1022 cm^−1^ and 995 cm^−1^ (Figure 2), indicative of lower structural order and less uniform hydration of the maltodextrin chains. This is associated with the lowest proportion of short maltooligosaccharides (DP 2–6) in its structure, the presence of DP8, a very high molecular weight (Mw), and an extremely broad polydispersity index (Pd) of 9.6, resulting in reduced solubility and WBC (Section 2.5, Section 2.6 and Section 2.7). This interpretation is consistent with the established understanding of the 1047/1022/995 cm^−1^ bands in starch-containing materials, as described by van Soest et al. [29] and Pozo et al. [30].

### 2.5. Non-Carbohydrate Components of Maltodextrin and the Profile of Maltooligosaccharides

Upon examining the non-carbohydrate constituents of the maltodextrins obtained, it was noted that maltodextrins derived from the starch of yellow potato varieties exhibited a protein content comparable to those derived from the starch of purple and red potato varieties (Table 3). It is important to highlight that a portion of the measured protein content also originates from the enzyme introduced during enzymatic hydrolysis in the production of maltodextrins, with an identical quantity of enzyme added to each sample. Although the enzyme is inactivated, due to the solubility of the resulting maltodextrins, the inactivated enzyme is not removed from the maltodextrin sample. Consequently, part of the detected protein may be attributed to the enzyme. Maltodextrins from the starch of yellow ‘Tajfun’ potatoes and red-fleshed ‘Magenta Love’ potatoes exhibited identical fat content. Among the analyzed maltodextrins, the highest fat content was observed in the maltodextrin derived from the starch of ‘Blue Star’ purple-fleshed potatoes, while the lowest fat content was found in the ‘Violetta’ variety, also a purple-fleshed potato (Table 3). Generally, the ash content in maltodextrins obtained from the starch of purple-fleshed and red-fleshed potatoes was approximately 28% higher than that in maltodextrins obtained from the starch of light-fleshed potato varieties. This was reflected in the phosphorus content, which was 28% higher in maltodextrins obtained from the starch of potatoes with colored flesh compared to those from the starch of yellow potato varieties (Table 3). The elevated ash and phosphorus content in maltodextrins from the starch of potatoes with colored flesh is attributable to the high levels of these components in the starches themselves [23]. Maltodextrin derived from the starch of purple-fleshed ‘Blue Star’ potatoes exhibited the highest ash and phosphorus content, whereas maltodextrin derived from the starch of the light-fleshed ‘Lord’ variety exhibited the lowest ash and phosphorus content (Table 3).

The maltooligosaccharide profiles of the maltodextrins examined (Table 4) exhibited distinct variations attributable to the botanical origin of the starch and the molecular characteristics of the maltodextrins. The lowest concentrations of all identified oligosaccharides were observed in maltodextrin derived from the yellow Tajfun potato variety (32.68 g/100 g d.m.). For the other varieties, this value ranged from 56 g/100 g d.m. to 70 g/100 g d.m. (Table 4). These findings are particularly noteworthy in relation to the dextrose equivalent (DE) values (Table 1). Although the maltodextrin from the Tajfun potato variety was characterized by a high DE (18), its maltooligosaccharide profile indicated a low proportion of short chains compared to the MV sample, which had the same DE but a different maltooligosaccharide composition—a high proportion of short chains (Table 1 and Table 4). This trend demonstrates that the DE value alone does not accurately reflect the actual degree of polymerization (DP) distribution, as previously mentioned, and cannot serve as the sole indicator of functional properties. Similar observations have been documented in the literature, including by Takeiti et al. [2] and Wang and Wang [11]. Consequently, two maltodextrins with similar DE values may exhibit significantly different maltooligosaccharide compositions, a finding that has also been corroborated in this study (Table 1 and Table 4).

Maltodextrins with a higher concentration of short oligosaccharides, particularly those derived from the starch of the colored potato varieties Violetta, Blue Star, and Magenta Love, demonstrated nearly complete solubility and elevated water-binding capacity (Section 2.7) values (Table 4). The maltodextrin from the Magenta Love variety exhibited the highest WBC, measuring 22.46 g water/g dry matter (d.m.) at 25 °C. Conversely, the Tajfun variety, which was characterized by the lowest total oligosaccharide content, exhibited the lowest solubility, ranging from 73 to 86%, as well as the lowest WBC value of 4.97 g water/g d.m., as presented in Section 2.7. This observation aligns with existing literature, which highlights that shorter maltooligosaccharides enhance the accessibility of hydroxyl groups, thereby increasing both water absorption capacity and solubility [7,31,32]. Additionally, shorter chains offer the advantage of reduced structural entanglement, facilitating water diffusion and promoting faster swelling of maltodextrins [3,7,31].

Notably, maltodextrin from the Lord variety, despite being derived from light-fleshed potatoes, exhibited a maltooligosaccharide profile akin to that of colored varieties (Table 4). This similarity directly accounts for its high solubility and favorable WBC, which markedly contrasts with the maltodextrins derived from the yellow starch of the Tajfun potato variety, as presented in Section 2.7. A comparison of the maltooligosaccharide profile with molecular weight parameters (Mn, Mw, and Pd), presented in Section 2.6 clearly indicates that the content of short oligosaccharides (DP1–DP6) is the primary determinant of both the average molecular weights and the breadth of the mass distribution (polydispersity) of the maltodextrins under investigation. Samples enriched with short oligosaccharide chains, such as Violetta, Blue Star, and Magenta Love, are characterized by lower Mn and Mw values and a low Pd (1.3–1.6), signifying a narrow and homogeneous molecular weight distribution in these preparations. This molecular structure promotes easy hydration, high solubility, and enhanced water-binding capacity. This tendency is consistent with the studies of other authors [7,31] who showed a correlation between the low molecular weight of maltodextrins and their increasing water-binding capacity and solubility.

In contrast, maltodextrin derived from the Tajfun potato variety, which exhibits the poorest degree of polymerization (DP) profile in terms of short oligosaccharide fractions and is the sole sample containing detectable maltooktaose (DP8) (Table 4), demonstrated a notably high molecular weight (Mw) of 24.35 × 10^3^ g/mol and an exceptionally broad mass distribution (polydispersity index, Pd = 9.6), presented in Section 2.6. This combination suggests the presence of a mixture of both short and very long chains, which significantly compromises the functional properties of this maltodextrin (Section 2.7), resulting in low solubility, reduced water-binding capacity (WBC), and a broad particle size distribution, as evidenced by the particle size analysis (Table 2). Ultimately, this relationship confirms that it is the actual DP distribution and the resulting Mn/Mw/Pd ratios, rather than the dextrose equivalent (DE) value itself, that determine the technological properties of maltodextrins [2,7,11,13,26,31,32]. By comparing the data from the particle size analyses (Table 2 and Table 4) with the sugar profiles, additional relationships can be discerned. Maltodextrins with a higher proportion of small particles (Violetta, Magenta Love, >95% of particles < 30 μm) are characterized by both a higher oligosaccharide content and enhanced solubility. This facilitates water access to the interior of the particles and promotes rapid swelling. Conversely, maltodextrin derived from the yellow potato variety Tajfun, which has the lowest proportion of particles < 30 μm (75.5%) and the highest SPAN (2.86—high heterogeneity; Table 2), simultaneously exhibited the lowest oligosaccharide content (Table 4) and the poorest functional properties (Section 2.7). This corroborates the findings of Siemons et al. [26], who reported that particle size distribution is a key factor determining the functional properties of maltodextrins, as it influences the interaction with water and diffusion kinetics. The mineral content (ash and phosphorus) was the highest in maltodextrin from the Blue Star potato variety, which also had one of the highest total maltooligosaccharide contents (Table 3 and Table 4). This may be attributed to the higher proportion of amylose and its phosphorylated forms in the starch of colored potatoes, as confirmed by Gumul et al. [23], Krystyjan et al. [24], and Chen et al. [33]. A higher phosphorus content promotes increased hydrophilicity of starch and its hydrolysis products.

### 2.6. Structural Parameters and Molecular Weights of Maltodextrins

**Figure 4:**
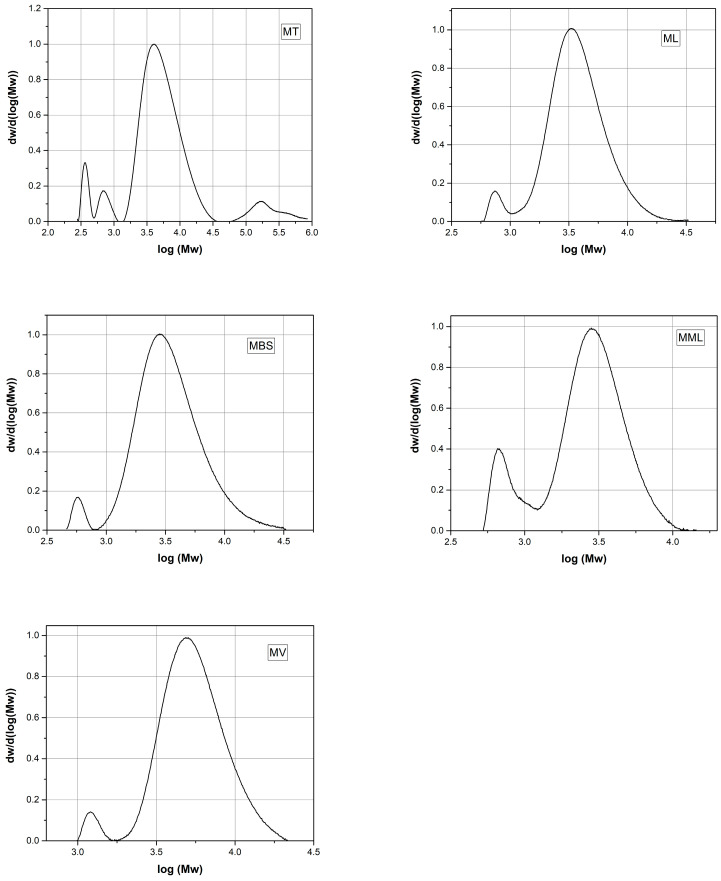
Differential molecular weight distributions of the maltodextrins studied.

Structural analysis of maltodextrins derived from various potato varieties revealed significant hydrolysis of starch polysaccharide chains during production. The number-average molecular weight and weight-average molecular weight of maltodextrins MBS, ML, and MV exhibit considerable similarity, suggesting that the uniformity of the raw material and the dextrinization process results in samples with analogous structural properties. Notably, the dispersion coefficients of these samples also demonstrate substantial similarity, and the molecular weight distribution is comparable. This observation indicates the presence of two high-molecular-weight fractions (exceeding 1000 g/mol), with fraction 1 (logMw = 3–3.25) comprising only a minor portion of the total sample mass. In both instances, the high-molecular-weight fraction (logMw = 3.25–4.25) predominates. The MV maltodextrin displayed a similar differential molecular weight distributions curve shape, characterized by a bimodal distribution in which the high-molecular-weight fraction is clearly dominant. However, for MML and MBS maltodextrins, the average molecular weight parameters were marginally lower than those observed in the MV system (Table 5; Figure 4).

Maltodextrin MT, however, demonstrates distinct characteristics. In this instance, the structural parameters (Mn, Mw, and Pd) and differential molecular weight distribution all varied. The weight-average molecular weight of this maltodextrin was more than four times greater (24.35 kg/mol) than that of the other systems. In contrast, the Mn value remained at a level comparable to that of all other maltodextrins. This resulted in a significant increase in the dispersion of maltodextrin. This parameter is 9.6, indicating that the sample contains a broad spectrum of oligo- and polysaccharide chains up to weight-average molecular weights of 10^−6^ g/mol, which are values similar to those of native potato starch. Concurrently, with such high dispersion, this sample exhibited significant multimodality in the form of four distinct fractions: two low-molecular-weight fractions (log Mw = 2.5–3), a dominant fraction in the range of log Mw = 3.2–4.5, and a polydisperse high-molecular-weight fraction (range log Mw = 4.7 to 6) (Table 5, Figure 4). Similar results regarding the multimodality of the distributions were obtained in the study by Avaltroni et al. [31]. Given the method of preparation of all samples, the explanation for this phenomenon must be based on the structural differences in the raw material, specifically potato starch obtained from the MT variety. These differences likely arise from the higher average molecular weight of this starch and possibly also from a different fractional distribution in the raw material concerning the chains, which are predominantly linear (amylose) and branched (amylopectin) [23].

For all samples analyzed, the maximum value of the dominant peak was observed to range between log Mw 3.4 and 3.7, corresponding to a degree of polymerization between 21 and 23, assuming a molecular weight of 162 for an anhydroglucose unit. These findings suggest a lower degree of depolymerization of the starch chains compared to commercial preparations, where polysaccharides with a degree of polymerization in the range of 10–12 are predominant [34].

### 2.7. Water-Binding Capacity (WBC) and Solubility (S) of Maltodextrins

The water-binding capacity at 25 °C for maltodextrin derived from Lord potato starch was 2.5 times greater than that of Tajfun. Among the maltodextrins obtained from the starch of colored-fleshed potatoes, the maltodextrin from the purple-fleshed Blue Star variety demonstrated the lowest water-binding capacity (Table 6).

Conversely, maltodextrin from Violetta purple-fleshed potatoes exhibited a water-binding capacity six times greater than that of maltodextrin from potatoes of the same flesh color. Maltodextrin from red-fleshed potatoes (Magenta Love) exhibited the highest water-binding capacity (Table 6). At 40 °C, no water-binding capacity was observed for the analyzed maltodextrins, as they were nearly 100% soluble. In contrast, the solubility at 25 °C was lowest in maltodextrins obtained from the starch of yellow-fleshed Tajfun potatoes, corresponding to their relatively low water-binding capacity. The remaining maltodextrins exhibited 100% solubility at room temperature (Table 6). Maltodextrins obtained from sweet potato starch were characterized by solubility ranging from 64.73% to 99.82% [33]. These findings are consistent with those of the present study.

According to the methodology outlined by Li et al. [6], it was observed that at 25 °C, maltodextrin derived from the starch of yellow-fleshed Tajfun potatoes demonstrated the lowest water-binding capacity. Conversely, maltodextrin sourced from ‘Lord’ potatoes of the same flesh color exhibited a water-binding capacity nearly eight times greater than that of the maltodextrin from ‘Tajfun’ potatoes (Table 7). 

Maltodextrin obtained from the starch of Blue Star variety potatoes with purple flesh was characterized by a low water-binding capacity at 25 °C, consistent with the results from the initial water-binding capacity measurement method. At 40 °C, the maltodextrin from the yellow-fleshed Tajfun variety recorded the lowest water-binding capacity (Table 7). Maltodextrins from ‘Lord’ variety potatoes of the same flesh color exhibited a water-binding capacity almost three times higher than that of the maltodextrins from Tajfun potatoes (Table 7). For maltodextrins from Magenta Love potatoes with red flesh, the water-binding capacity was approximately 78% higher than that of maltodextrins from Tajfun potatoes and nearly 30% lower than that of maltodextrins from Lord potatoes. Maltodextrins from Blue Star potatoes with purple flesh exhibited the highest water-binding capacity, exceeding that of maltodextrins from Tajfun potatoes by more than four times (Table 7). At 25 °C, maltodextrin from Magenta Love red-fleshed potatoes showed no water-binding capacity, and at both 25 and 40 °C, maltodextrin from Violetta purple-fleshed potatoes exhibited no water-binding capacity. Under these conditions, the maltodextrins demonstrated 100% solubility. The maltodextrin from the starch of yellow-fleshed Tajfun potatoes exhibited the lowest solubility at both tested temperatures (Table 7). At both 25 and 40 °C, the solubility of maltodextrin from the yellow-fleshed Lord variety and the purple-fleshed Blue Star variety remained at a similar level. The solubility of maltodextrin from Lord potatoes was approximately 98.3%, which was higher than that of maltodextrin from Tajfun potatoes by approximately 35% at 25 °C and nearly 15% at 40 °C. The solubility of maltodextrin from Blue Star potatoes was approximately 97.5%. Compared to maltodextrin from the Tajfun potato variety, maltodextrin from the Blue Star potato variety exhibited approximately 33% higher solubility at 25 °C and nearly 15% higher at 40 °C (Table 7). In contrast, maltodextrin from Magenta Love potatoes with red flesh demonstrated approximately 14% higher solubility at 40 °C than maltodextrin from Tajfun potatoes with yellow flesh (Table 7).

Upon comparing the water-binding capacity and solubility results at two distinct temperatures (25 and 40 °C) using two methodologies [6,35], it can be concluded that there are no significant differences in the water-binding capacity and solubility of maltodextrins derived from light-fleshed and colored potato starch (samples: ML, MML, MV, and MBS). An exception to this observation is the maltodextrin derived from the starch of light-fleshed potatoes of the Tajfun variety (MT), which demonstrated the lowest water-binding capacity and solubility relative to the other maltodextrins examined, irrespective of the parameters measured and the analytical methods employed (Table 6 and Table 7).

It should be emphasized that the high solubility of maltodextrins is a key application feature in food technology, particularly for the microencapsulation of bioactive compounds. Maltodextrins derived from red-purple potato starch have significantly greater solubility and water absorption capacity than maltodextrins derived from yellow potato starch, and therefore may be more suitable as a wall material (external phase) in encapsulation. High solubility (100%), low viscosity at high solids concentration, low relative cost, neutral taste, and aroma of such maltodextrins can guarantee a good material as a wall material (external phase) in encapsulation. The wall material acts as a physical barrier to block light, oxygen, water, etc., so it can store some bioactive substances that we want to protect from adverse external environmental conditions during technological processes [19,20,21].

### 2.8. Viscoelastic Properties of Maltodextrins

**Figure 5:**
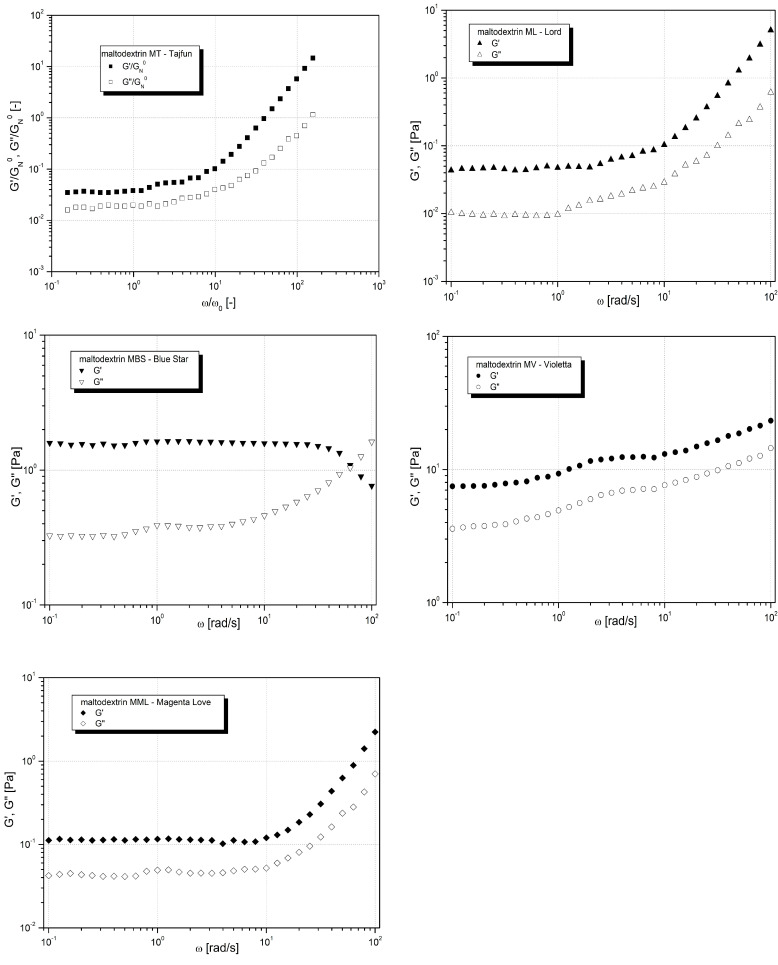
Mechanical spectrum of potato maltodextrins.

Figure 5 presents the experimental values of the storage modulus G′ and loss modulus G″ as a function of the oscillation frequency ω for the tested potato maltodextrins, i.e., their so-called mechanical spectra.

Over the entire analyzed range of oscillation frequencies, potato maltodextrins exhibit elastic properties over viscous ones, with higher values of the storage modulus G′ over the loss modulus G″.

Table 8 presents the values of rheological parameters obtained directly from rheometric measurements described by equations from (1) to (13).

The data in Table 8 indicate the following:

- The total network elasticity G_e_ of potato maltodextrins varies considerably, from very low values for maltodextrins isolated from yellow potato varieties Tajfun (MT) and Lord (ML) to high values for maltodextrins obtained from colored potatoes, i.e., red Magenta Love (MML) and purple Blue Star (MBS) and Violetta (MV). The difference between the lowest total network elasticity value for the Tajfun variety (G_e_ = 0.0194 Pa) and the highest value obtained for the Violetta variety (G_e_ = 7.480 Pa) is as much as 385 times.
- The susceptibility in the equilibrium state of energy J_e_, and therefore the possibility of storing energy, is the highest for maltodextrin obtained from yellow potatoes of the Tajfun variety (MT).
- The values of the plateau viscoelastic modulus G_N_^0^ are similarly varied as the values of the total network elasticity G_e_. They show that the potato maltodextrin samples tested are media with a structure exhibiting behavior typical of viscoelastic quasi-solids. The G_N_^0^ modulus values indicate that the structure’s cross-linking is strongest for maltodextrin isolated from purple potatoes of the Violetta (MV) variety, and for this maltodextrin, aging processes occur more slowly over time.
- The susceptibility of the structure to J_N_^0^ cross-linking reached the highest values for maltodextrins obtained from yellow potato varieties, i.e., the Tajfun (MT) variety and the Lord (ML) variety, at the level of 1.786 1/Pa and 1.815 1/Pa, respectively, which means that these maltodextrins have such network entanglements that suppress all types of long-range configurational rearrangements.
- The highest values of the network vibration damping coefficient k were achieved for maltodextrins isolated from yellow potato varieties, namely Tajfun (MT) and Lord (ML). In practice, this means that the network formed by these maltodextrins is resistant to external vibrations, and therefore media containing these maltodextrins will exhibit behavior typical of soft gels, while media containing maltodextrins from the Blue Star (MBS) and Magenta Love (MML) potato varieties will exhibit behavior typical of hard gels, with low values of the k coefficient.
- The Newtonian viscosity under steady-state flow conditions h_0_ has the highest value for maltodextrin isolated from a purple potato variety, namely the Violetta (MV) variety, which means that the flow capacity of the set of elements enclosed by the minimum number of network nodes for this particular maltodextrin is the lowest. Interestingly, maltodextrin obtained from the second purple potato variety, Blue Star (MBS), had a h_0_ viscosity five times lower, so maltodextrin solutions from the Violetta variety will allow for obtaining media characterized by higher gel stiffness.
- The cross-link density ω_0_ values of the tested maltodextrins indicate when the viscoelastic plateau region, i.e., the highly elastic state, ends. This state ends earliest for maltodextrin obtained from the purple Blue Star potato variety (MBS)—the lowest cross-link density value ω_0_ = 0.068 rad/s. This cross-link density value also indicates the distances between each node in the network being formed. In the case of maltodextrin from Blue Star potatoes, network nodes will appear every 0.068 rad/s, which in practice means that the network will be very densely woven. The opposite situation occurs in the case of two other potato varieties, namely maltodextrins isolated from yellow potato varieties, i.e., Tajfun (MT) and Lord (ML), for which the cross-linking density was 0.640 rad/s and 0.535 rad/s, respectively.
- The width of the viscoelastic plateau L has the highest values, equal to 28.866 and 12.696, respectively, for maltodextrins obtained from yellow potato varieties, i.e., Tajfun (MT) and Lord (ML), suggesting greater polydispersity in relation to maltodextrins from other potato varieties.
- The values of the average molecular weights for Me entanglement and Mc cross-linking do not have the same order of magnitude and confirm the different polydispersity of the tested maltodextrins.
- The mesh size ζ is the largest for maltodextrins obtained from yellow potato varieties, i.e., Tajfun (MT) and Lord (ML), 199.663 nm and 200.744 nm, respectively, while it is 2 times smaller for maltodextrin from Blue Star (MBS) and Magenta Love (MML) potatoes, and almost 5 times smaller for maltodextrin from Violetta (MV) potatoes.

Analysis of the discussed rheological parameters allows us to conclude that maltodextrins isolated from the yellow potato varieties Tajfun (MT) and Lord (ML) will form a soft, elastic gel with a structure that is quite resistant to external mechanical vibrations. This is because these maltodextrins enable the production of solutions with relatively low viscosities while simultaneously exhibiting a relatively high susceptibility to cross-linking, which creates a network that is not very dense but flexible. In the case of maltodextrins isolated from the red potato variety Magenta Love (MML) and the purple potato variety Blue Star (MBS), the situation is reversed. These maltodextrins will produce solutions with high viscosities and, at the same time, low susceptibility to cross-linking. This network will be quite densely woven, but compared to maltodextrins from yellow potato varieties, it will be significantly less flexible, thus exhibiting behavior typical of rather hard gels. Maltodextrin obtained from the purple potato variety Violetta (MV) is characterized by the ability to produce solutions with extremely high viscosity values, but also a structure characterized by low susceptibility to cross-linking. This structure will, however, be able to dampen external mechanical vibrations, thanks, in part, to the small mesh size of the network created and its high elasticity.

**Figure 6:**
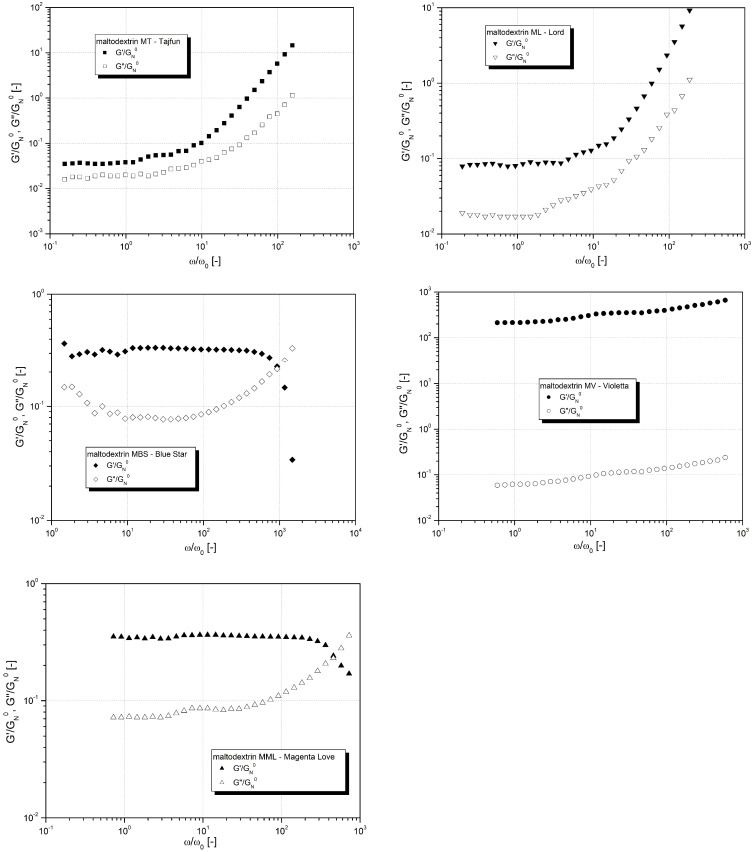
Reduced curves of potato maltodextrins.

The graph in Figure 6 shows reduced curves in the coordinate system G′/G_N_^0^ = *f*(ω/ω_0_) and G″/G_N_^0^ = *f*(ω/ω_0_), assessing the superposition of the experimental curves obtained for the tested potato maltodextrins.

The reduced curves of all tested potato maltodextrin samples, both G′/G_N_^0^ = *f*(ω/ω_0_) and G″/G_N_^0^ = *f*(ω/ω_0_), are not subject to superposition, indicating a varying contribution of elements shaping both their elastic and viscous properties. This indicates a very diverse scale of dissipative phenomena across the analyzed range of oscillation frequencies for each of the tested potato maltodextrins.

**Figure 7:**
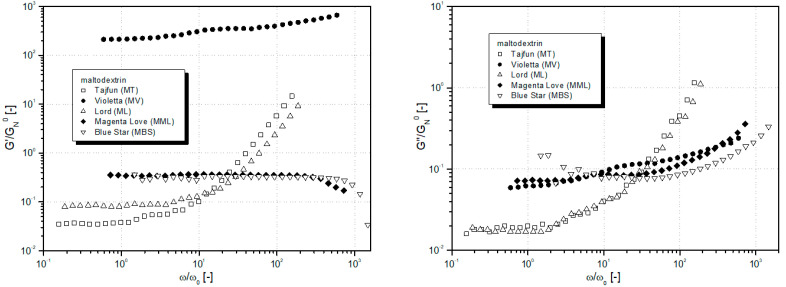
Reduced curves of potato maltodextrins—comparison.

For a better comparison of the viscoelastic properties of the tested maltodextrins, it was decided to compare the differences in the share of viscous and elastic elements shaping the internal structure of these maltodextrins—the results are presented in the graph in Figure 7.

The data presented in the graph in Figure 7 show the following:

- The elastic elements shaping the internal structure of the yellow potato varieties Tajfun (MT) and Lord (ML) are very similar—there is a certain superposition of the reduced curves G′/G_N_^0^ = *f*(ω/ω_0_), which is also reflected in the values of the rheological parameters (see Table 8).
- Practically complete superposition of the reduced curves G′/G_N_^0^ = *f*(ω/ω_0_) is observed for two potato varieties, each of which has a different flesh color, i.e., for maltodextrin obtained from the red potato variety Magenta Love (MML) and the purple potato variety Blue Star (MBS)—this means that the share of elastic elements shaping their internal structure is practically the same, with very similar values of the G_N_^0^ and G_e_ parameters (Table 8).
- In the case of maltodextrin from the purple potato variety Violetta (MV), the elastic elements shaping its internal structure are definitely different from those of other potato varieties, explaining what is likely the highest value of the viscoelastic modulus of the plateau G_N_^0^ (Table 8).
- Viscous elements illustrated in the form of reduced curves G″/G_N_^0^ = *f*(ω/ω_0_) show the similarity of these elements in shaping the internal structure of maltodextrins isolated from two yellow potato varieties Tajfun (MT) and Lord (ML): full superposition of reduced curves in the entire range of oscillation frequencies and similar values of viscosity η_0_ and cross-linking density ω_0_ (Table 8).
- A very high similarity of the viscous elements in shaping the internal structure was observed for three maltodextrins with colored flesh, i.e., for red-fleshed potatoes of the Magenta Love (MML) variety and purple varieties Violetta (MV) and Blue Star (MBS). There is no complete superposition of the curves; these curves are similar to each other, as are the values of their rheological parameters (Table 8).

**Figure 8:**
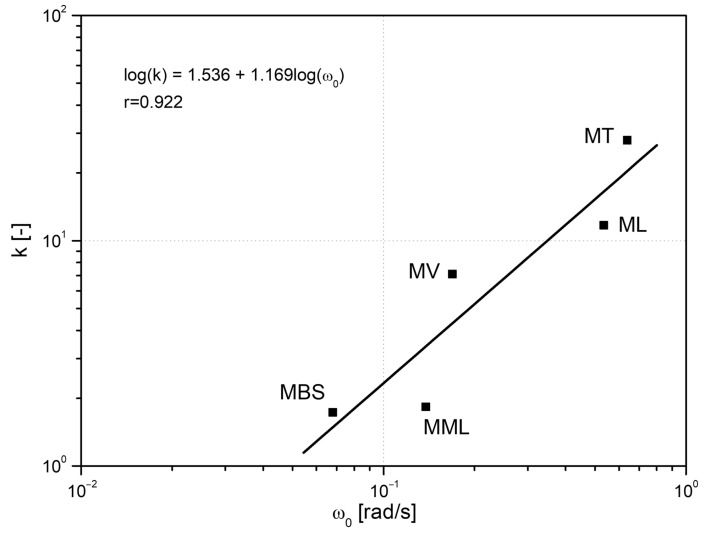
Correlation relationship between rheological parameters k and ω_0_.

Taking into account the behavior of aqueous solutions of potato maltodextrins as soft and hard gels, it was decided to present a correlation between two rheological parameters that represent both the elastic properties—parameter k—and the viscous properties—parameter ω_0_ of the tested maltodextrin solutions (Figure 8).

The graph in Figure 4 confirms the strong, positive correlation between these two rheological parameters, which may indicate a similarity in the elementary structural units of the isolated potato maltodextrins.

Summarizing the considerations on the viscoelastic properties of the isolated potato maltodextrins, they can be ranked according to their behavior as hard and soft gels, the series is as follows:


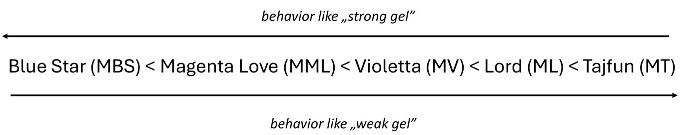


Maltodextrins isolated from the yellow potato varieties Tajfun (MT) and Lord (ML) produce media exhibiting behavior typical of soft gels. The softest gels are obtained using maltodextrin isolated from the Tajfun (MT) variety. This maltodextrin has the highest weight-average molecular weight (M_w_) of 24.35·10^3^ g/mol and the highest polydispersity index (PD) of 9.6 among all maltodextrins tested. The carbohydrate profile of this maltodextrin shows the lowest amount of maltooligosaccharides, at 32.68 g/100 g.s.s. The content of all carbohydrates in this profile—except maltoheptaose—is lower for this particular maltodextrin than for other maltodextrins. The maltoheptaose content is significantly higher than for other maltodextrins, at 7.14 g/100 g s.d. (Table 4 and Table 5). It is maltoheptaose that is the oligosaccharide that can increase the elasticity and flexibility of the structure of formed gels, especially in bakery and confectionery products, giving them the so-called desired chewiness—it should therefore create soft, easily moldable gels, as shown by the rheological tests.

At the other end of the spectrum are maltodextrins, which will lead to solutions exhibiting behavior typical of hard gels and can be obtained from the purple potato variety Blue Star (MBS) and the red variety Magenta Love (MML). The total maltooligosaccharide content of these two maltodextrins is the highest in their carbohydrate profile, while the maltoheptaose content—responsible for elastic properties—is the lowest.

The hardest gels are obtained using maltodextrin from the Blue Star variety (MBS)—it is for this maltodextrin that the high-elastic state ends first and a densely woven network with the lowest cross-linking density ω_0_ is formed. Gels obtained using maltodextrin from Blue Star potatoes (MBS) exhibit greater hardness than those derived from Magenta Love potatoes (MML) because Blue Star maltodextrin (MBS) is characterized by higher weight and number-average molecular weights and greater polydispersity than Magenta Love maltodextrin (MML), and additionally contains a higher percentage of fat as a non-carbohydrate component—at the level of 0.51%. This small difference in fat content between these two maltodextrins—just 0.08%—determines that Blue Star potato maltodextrin (MBS) will form harder gels. Higher fat content increases stiffness and storage modulus G′ values—(Figure 5)—making the resulting gel more “full”, “compact” and often harder, which can reduce its elastic properties [36,37]. Therefore, with the very comparable carbohydrate profile of these two maltodextrins, it was the fat content that proved decisive, shifting the gel hardness toward the maltodextrin from the purple Blue Star potato variety (MBS).

To the best of the authors knowledge, the rheological properties of maltodextrin solutions have not been analyzed using phenomenological rheology methods. Therefore, it is very difficult to compare the obtained results with those of other authors. Nevertheless, one study examining the effect of maltodextrins on the rheological properties of potato starch pastes and gels is noteworthy. It was found that medium-saccharide maltodextrins (DE glucose equivalent = 18.4) reduce the storage and loss modulus the most compared to low- and high-saccharide maltodextrins, which is directly related to the degree of polymerization of the maltooligosaccharides present in them. Maltodextins isolated from the potato varieties tested in this study belong to the group of medium-saccharide maltodextrins with a DE glucose equivalent ranging from 15% to 18%. According to Juszczak et al. [38], it is these maltodextrins that have the greatest impact on the gelatinization characteristics and rheological properties of the resulting pastes and gels. Based on this data, the authors of this study demonstrated something more: that the potato variety from which maltodextrin was isolated may determine the behavior (hard or soft) of gels prepared with it.

## 3. Materials and Methods

### 3.1. Materials

#### 3.1.1. Characteristic of Potatoes

Red and purple-fleshed potatoes of varieties Magenta Love, Blue Star and Violeta and yellow-fleshed potatoes of varieties Lord and Tajfun were grown in the year 2023 in a field nursery at the Department of Environmental Protection and Organic Farming in Spišská Belá (Slovakia). All of the above-mentioned potato varieties are edible (*Solanum tuberosum* L.). Among them, those with traditional yellow flesh (Lord and Tajfun varieties), characterized by both yellow flesh and yellow skin, are very early and early maturing varieties. Other varieties are colored varieties, such as Violetta, with dark purple skin and dark purple flesh, and a high anthocyanin content (195 mg of cyanidin-3 glycoside/100 g dry matter); Magenta Love with intensely red flesh and intensely red skin, also containing a large amount of anthocyanins (240 mg of cyanidin-3-glycoside/100 g dry matter); and Blue Star with purple-blue flesh and purple-blue skin (anthocyanin content—96 mg of cyanidin-3-glycoside/100 g dry matter). The Tajfun, Lord, Blue Star, Violetta, and Magenta Love varieties contained the following amounts of starch: 17.3; 17.00; 15.13; 14.80 and 15%, respectively.

#### 3.1.2. Isolating Starch

The starch was extracted utilizing a laboratory procedure as delineated by Wischmann et al. [39]. Initially, a batch of 10 kg of potatoes was meticulously washed under running water, from which 2 kg of undamaged potatoes were periodically selected. This portion was ground into a uniform pulp and transferred in increments onto cheesecloth positioned on a Büchner funnel, where manual starch washing commenced. The washing process was terminated when the filtrate collected beneath the sieve exhibited a negative reaction with Lugol’s solution. A negative iodine color reaction permitted the continuation of starch extraction from subsequent pulp batches. Upon completion of washing the entire batch of crushed potatoes, the starch collected in the beaker was allowed to settle with the cell sap for approximately 8 h. The supernatant was then carefully decanted, after which the beaker was replenished with clean water, stirred, transferred to a centrifuge tube, and centrifuged. The resultant slurry was collected, transferred to a beaker, covered with water, and stirred. The supernatant was decanted once more, water was added again, the mixture was stirred, and the supernatant was left until it became completely clear. The purified starch was then transferred to filter paper and air-dried at 23 °C, subsequently ground in an RM 200 mill (Retsch GmbH, Haan, Germany), sieved through a laboratory sieve AS 200 (Retsch GmbH, Haan, Germany) with a mesh size of 0.2 mm, and stored in jars for further analysis.

#### 3.1.3. Production of Maltodextrins

Maltodextrins were synthesized using an IKA LR 1000 control laboratory reactor (IKA-Werke GmbH & Co. KG, Staufen im Breisgau, Germany). A quantity of starch (220 g, dry weight basis) was accurately measured and transferred into the reaction chamber, followed by the addition of 440 cm^3^ of distilled water and 120 mg of calcium chloride. The chamber was subsequently sealed with a lid. The pH of the starch suspension was adjusted to 6 using a 0.1 M hydrochloric acid solution. The stirring mechanism was activated, set to a speed of 150 rpm, and the temperature was maintained at 25 °C. Concurrently, a 50 µL sample of the BAN 480 L enzyme preparation (Novozymes, Bagsværd, Denmark) was heated to 84 ± 1 °C and held at this temperature for 20 min. Following this period, the starch slurry was rapidly heated to 95 °C, maintaining this temperature for an additional 20 min to ensure enzyme inactivation. The resultant hydrolysates were then transferred into Petri dishes and subjected to drying in a BMT Venticell 55 dryer (BMT, Brno, Czech Republic) with air circulation at 40 °C for 3–4 days until a moisture content of 10–15% was achieved. The final preparation was ground using an RM 200 mortar mill (Retsch GmbH, Haan, Germany) and sieved with an AS 200 laboratory sieve (Retsch GmbH, Haan, Germany) featuring a mesh size of 0.2 mm [40].

### 3.2. Methods

#### 3.2.1. Determination of the Dextrose Equivalent (DE)

The dextrose equivalent (DE) value was determined using the Schoorl-Regenbogen method, as specified in the Polish standard PN-78/A-74701 (1978) [41]. This method involves the iodometric determination of the excess of copper (II) ions that have not reacted with sugars during the reduction reaction. The quantity of copper was ascertained based on the iodide released from potassium iodine, which was subsequently titrated with sodium thiosulfate.

#### 3.2.2. Maltodextrin Particle Size Distribution and Polydispersity Index (PDI–SPAN)

The analysis of maltodextrin was conducted utilizing the Analysette 22 NeXT laser particle size analyzer (Fritsch, Idar-Oberstein, Germany). A sample weighing 0.1 g was accurately measured and subsequently dispersed in deionized water using a vortex mixer for 10 s. The measurements were carried out in accordance with the established standard operating procedure.

The polydispersity index was determined using the formula (D90 − D10)/D50, commonly referred to as SPAN. This index is also known as the spread index or width polydispersity index. The interpretation of SPAN values is as follows:

- A value of less than 0.8 indicates a very narrow distribution, approaching monodispersity; values between 0.8 and 1.2 suggest a narrow distribution with very good uniformity;
- Values ranging from 1.2 to 2.0 are indicative of an average distribution, typical for most food products;
- Values between 2.0 and 3.0 denote a wide distribution, reflecting high heterogeneity; and values exceeding 3.0 represent a very wide distribution, which is generally considered unacceptable.

#### 3.2.3. Scanning Electron Microscopy

Microscopic observations were conducted following the methodology outlined by Rosicka-Kaczmarek et al. [42]. The images were captured using a Jeol-JCM-6000 scanning electron microscope (Jeol, Akishima, Japan). The samples under examination were sputter-coated with gold in a vacuum environment (without the use of noble gases), and images were acquired at acceleration potentials ranging from 5 to 10 kV.

#### 3.2.4. Structure Analysis Using Fourier Transform Infrared Spectroscopy (FT-IR)

The Fourier transform infrared (FT-IR) spectra of the synthesized maltodextrins were obtained using an IRTracer-100 FT-IR spectrometer (Shimadzu Corporation, Kyoto, Japan) equipped with an ATR attachment. The measurements were conducted in transmittance mode over the spectral range of 600–4000 cm^−1^, with a resolution of 4 cm^−1^, at a temperature of 20 °C, and involved 40 scans.

#### 3.2.5. Content of Non-Polysaccharide Components

The protein content was determined in accordance with the AOAC [43] guidelines. Samples were placed in Kjeldahl flasks, to which a selenium mixture was added as a catalyst, followed by sulfuric acid. Mineralization was conducted until the solution attained a light green hue. Subsequently, the flask was cooled, and its contents were diluted by the gradual addition of water. The sample was then transferred to a Parnas-Wagner apparatus and treated with a 33% sodium hydroxide solution. Distillation was carried out for approximately 15 min, and the condensate was collected in a conical flask containing 0.05 mol/L sulfuric acid and a few drops of Tashiro indicator. Post-distillation, the unbound acid was titrated with 33% sodium hydroxide. The ash content of the sample was also determined following AOAC [43] procedures. Approximately 2 g of the sample was incinerated in a quartz crucible over a Bunsen burner and subsequently placed in a hot muffle furnace at 900 °C for about an hour until all carbon residues were eliminated. The total phosphorus content in starch was analyzed according to EN ISO 3946 [44]. A 0.5 g sample was mineralized in a 1:1 *w*/*w* mixture of sulfuric and nitric acids (15 mL). The sample was then diluted with water, and the pH was adjusted to 7 using an aqueous solution of 10 mol/L NaOH. The neutralized solution was treated with ammonium heptamolybdate (VI) tetrahydrate (4 mL) and 50 g/L of ascorbic acid (2 mL). The intensity of the absorption band was measured at 825 nm.

#### 3.2.6. Maltooligosaccharides Content by HPLC

A comprehensive qualitative and quantitative analysis of maltooligosaccharides was conducted using Shodex NH2P-50 series columns, following previously established methodologies with certain modifications. Initially, 0.1 g ± 0.0001 g of the samples were combined with 3 mL of ultrapure water and incubated in a shaking water bath at 58 °C for 45 min. Subsequently, the samples were cooled and subjected to centrifugation (3024× *g*, 20 min, 20 °C). Post-centrifugation, the supernatant was promptly decanted and filtered through a nylon syringe filter with a 0.2 µm pore size into HPLC measuring vials. The filtrate was analyzed for maltooligosaccharide content using a UHPLC+ Dionex UltiMate 3000 system (Thermo Fisher Scientific Inc., Waltham, MA, USA) equipped with a refractive index detector (Shimadzu, Kyoto, Japan) and an Asahipak NH2P-50 4E column (4.6 × 150 mm, 5.0 µm particle size; Shodex, Japan). Isocratic elution was performed using a 70/30 (*v*/*v*) acetonitrile/water mixture as the mobile phase. The flow rate was maintained at 1.0 mL/min, and the column temperature was set at 30 °C. Glucose, maltose, maltotriose, maltotetraose, maltopentaose, maltohexaose, maltoheptaose, and maltooctaose were identified by comparing their retention times with those of authentic standards. Quantification was achieved using the external standard method.

#### 3.2.7. Analyses of Average Molecular Weights and Their Distributions

The average molecular weights and distributions were evaluated through gel permeation chromatography (GPC) [45], a size-exclusion analytical technique employed to separate and characterize polymers and biomolecules according to their size or hydrodynamic volume. The system employed consisted of three columns: Ultrahydrogel-2000 (Waters Corporation, Milford, MA, USA), Ultrahydrogel-500 (Waters), and Ultrahydrogel-120 (Waters), arranged in series with a refractive index (RI) detector (Knauer, Berlin, Germany). A solution of 0.1 M NaNO_3_ and 0.02% NaN_3_ in water served as the eluent. The flow rate was maintained at 0.6 mL/min, and the sample volume was 100 mL. The concentration of the samples was approximately 5 mg/mL. Sample preparation involved dissolution in 1 M NaOH followed by neutralization with 1 M HCl, using phenolphthalein as an indicator. Calibration was conducted using pullulan standards (Shodex, Tokyo, Japan).

#### 3.2.8. Determination of Water-Binding Capacity and Water Solubility

Determination of water-binding capacity and water solubility at 25 °C and 40 °C was carried out according to the method of Li et al. [6]. The test sample, with a dry weight of 0.15 g, was accurately weighed into a pre-weighed centrifuge tube, to which 10 cm^3^ of distilled water was subsequently added. The prepared sample was then subjected to a water bath equipped with a shaker (MEMMERT WB 22, Schwabach, Germany) at the specified temperature for the determination process. The sample was heated for one hour, with the contents of the tube being mixed every 10 min using a vortex mixer. Following the heating process, the sample was allowed to cool and was then centrifuged for 30 min at 1070× *g*. The supernatant obtained was carefully decanted into a pre-weighed weighing flask and subjected to evaporation and drying at a constant temperature of 105 °C in an SML 350 oven (Kraków, Poland) until a constant weight was achieved. After cooling in a desiccator, the weighing bottle was weighed to ascertain the solubility of the sample. The precipitate, comprising the sample and the water absorbed by it, was weighed to determine the water-binding capacity of the sample.

#### 3.2.9. Determination of Maltodextrin Solubility and Water-Binding Capacity

Determination of maltodextrin solubility and water-binding capacity was carried out according to the Leach method [35]. A sample of maltodextrin (1 g, dry weight basis) was accurately weighed with an accuracy of ±0.0001 g into a tared 100 cm^3^ centrifuge tube, followed by the addition of 70 cm^3^ of distilled water. The prepared sample was then agitated in a water bath with a shaker (MEMMERT WB 22, Schwabach, Germany) at temperatures of 25 °C and 40 °C for 30 min at a stirring speed of 200 rpm. Upon completion of the heating period, the stirrer was rinsed with distilled water, the sample was cooled, and its mass was adjusted to 80 g with distilled water. The resulting paste was centrifuged using a high-speed brushless Centrifuge MPW-350 (Warsaw, Poland) for 15 min at 1360× *g*. The supernatant obtained post-centrifugation was utilized to determine solubility, while the residue remaining in the vessel was employed to assess water-binding capacity. For the determination of maltodextrin solubility, 20 cm^3^ of the supernatant was transferred to a weighing flask, which was then placed under a radiant lamp for 1 h. Subsequently, the samples were dried in a drying oven (SML 350, Kraków, Poland) at 130 °C for 1 h, and after cooling, the samples were weighed.

#### 3.2.10. Rheological Measurements

For each of the isolated potato maltodextrins, a 5% solution was prepared as follows: a sample of the test substance was measured, made up to 100 mL with water, and then mixed. This produced an aqueous solution with a predetermined percentage concentration, which is the weight/volume (*w*/*v*) concentration. This concentration is therefore the ratio of the mass of the solute to the volume of the solution, expressed as a percentage (%).

After preparing the solution, it was left to rest for 60 min at an ambient temperature of 25 °C. After this time, a sample of the maltodextrin solution was placed in the measuring system of the rotational rheometer and left to rest for 30 min at a constant temperature of 50 °C to achieve thermal and mechanical equilibrium.

The rheological properties of the tested maltodextrin solution were determined using a Physica MCR 301 rotational rheometer by Anton Paar in a cone-plate measuring system with a cone diameter of 50 mm, an angle of inclination of 1^0,^ and a distance between the measuring elements, i.e., a cone and a plate of 0.048 mm. Rheological tests were carried out, consisting of measuring the viscoelastic properties of the tested maltodextrin samples.

#### 3.2.11. Measurement of Viscoelastic Properties

The study of viscoelastic properties was carried out in dynamic conditions in the controlled strain mode, determining the mechanical spectra by measuring the values of the storage modulus G′ and the loss modulus G′. The tests were carried out in a wide range of oscillation frequencies ω from 0.1 rad/s to 100 rad/s, taking 10 measurement points for each decade. The relative strain value for the potato maltodextrin samples tested was determined in previous studies examining the range of linear viscoelasticity. For all potato maltodextrins analyzed, the relative strain value determined was 1%. The measurements were performed in triplicate, and the final result is their arithmetic mean.

Based on the rheometric measurements, certain coefficients describing the viscoelastic properties of the potato maltodextrins tested, called rheological parameters, were determined. These include [46,47,48]:

- The equilibrium modulus, i.e., the modulus of elasticity in a steady state G_e_, which is responsible for the total elasticity of the medium:

(1) $$G_{e}=\lim_{\omega \to 0}G^{\prime }$$

- Viscoelastic plateau modulus G_N_^0^, responsible for cross-linking of the structure. Its high values also indicate the possibility of slowing down the aging effects of the medium over time; for polydisperse media, isolated samples of maltodextrins can be considered as such.
- It is determined from the relationship:

(2) $${G_{N}}^{0}=\frac{4}{\pi }\int_{\omega_{min}}^{\omega_{max}}G^{\prime \prime }\left(\omega \right)d\ln\left(\omega \right)$$

- Newtonian viscosity in conditions of steady flow η_0_ characterizing the flow capacity of a set of elements closed with a minimum number of nodes of the biopolymer network, capable of individual movement:

(3) $$\eta_{0}=\lim_{\omega \to 0}\frac{G^{\prime \prime }}{{\left(\omega \right)}^{2}}$$

These three basic parameters obtained directly from rheometric measurements make it possible to additionally determine:

- The equilibrium compliance J_e_, which is a measure of the energy stored during a steady state of the biopolymer under low-stress conditions:

(4) $$J_{e}=\left({\frac{1}{G}}_{e}\right)$$

- Plateau compliance J_N_^0^, representing the strength with which the entanglements of the biopolymer network suppress all kinds of long-range configuration rearrangements:

(5) $$J_{N}^{0}=\left(\frac{1}{G_{N}^{0}}\right)$$

- Network vibration damping coefficient k, which indicates how resistant the created medium structure is to external vibrations:

(6) $$k=\frac{G_{N-}^{0}G_{e}}{G_{e}}$$

- Weighted average relaxation time τ_m_ equated with the longest relaxation time:

(7) $$\tau_{m}=J_{e}\cdot \eta_{0}$$

- Numerically average relaxation time τ_0_ equated with the shortest relaxation time:

(8) $$\tau_{0}=\frac{\eta_{0}}{{G_{N}}^{0}}$$

- Cross-linking density of the structure ω_0_, defining at the same time the end of the viscoelastic area of the plateau:

(9) $$\omega_{0}=\frac{1}{\tau_{0}}$$

- Dimensionless width of the viscoelastic plateau L, combining fast and slow dissipation processes, determining the degree of biopolymer polydispersion:

(10) $$L=\frac{\tau_{m}}{\tau_{0}}$$

- Average molecular weight by entanglement M_e_, which is the average molecular weight between the topological bonds of the biopolymer network, resulting from the physical (mechanical) entanglement of long biopolymer chains:

(11) $$M_{e}=\frac{\rho_{k}\cdot R\cdot T}{G_{N}^{0}}$$

- Average molecular weight at M_c_ cross-linking, which is the average molecular weight of biopolymer chains between successive nodes of the network, which can be chemical cross-links, crystalline regions, and even polymer complexes:

(12) $$M_{c}=\frac{\rho_{k}\cdot R\cdot T}{G_{e}}$$

where: ρ_k_—density of maltodextrin solution, R—universal gas constant, and T—the temperature during rheological tests.

- Mesh size of the resulting network ξ, which is one of the characteristic linear dimensions of the created network of viscoelastic material:

(13) ξ=kB·TGN013

where: k_B_—Boltzman constant.

Additionally, differences in the structure of the prepared 5% (*w*/*v*) were presented in the form of so-called reduced curves in the coordinate system G′/G_N_^0^ = *f*(ω/ω_0_) and G″/G_N_^0^ = *f*(ω/ω_0_), assessing the superposition of the obtained experimental curves. This allows us to determine the degree of variation in the contribution of elements shaping the viscous and elastic properties of the tested potato maltodextrins. The lack of superposition of reduced curves indicates a different scale of dissipation phenomena within the fast or slow dissipation processes, while the full superposition of reduced curves indicates a uniform type of structure formed.

#### 3.2.12. Statistical Analysis

The data were analyzed using a one-way analysis of variance (Duncan’s post hoc test at a significance level of 0.05). Pearson’s correlation coefficients were employed to evaluate the relationships between the analyzed parameters (Table S1—Supplementary Materials).

All calculations were conducted using Statistica 11.0 (StatSoft Inc., Tulsa, OK, USA).

## 4. Conclusions

In summary, maltodextrins derived from starch isolated from red- and purple-fleshed potatoes exhibited a higher prevalence of fine agglomerates in SEM analysis, with over 95% of particles measuring less than 30 µm, and a substantial content of low-molecular-weight maltooligosaccharides (including glucose, maltose, maltotriose, maltotetraose, maltopentaose, and maltohexaose) compared to those obtained from the starch of light-colored potatoes, particularly the Tajfun variety. Regarding the non-carbohydrate components, maltodextrins from red and purple potato starch demonstrated a 28% higher ash and phosphorus content and an equivalent protein content relative to maltodextrins derived from yellow potato starch. FT-IR analysis indicated a greater degree of order and a more coherent hydrogen-bond network in maltodextrins from colored potatoes, consistent with the DP profiles (maltooligosaccharides DP1 to DP6) and parameters such as a lower Mn/Mw ratio and low Pd. Conversely, maltodextrin from the Tajfun yellow potato variety exhibited lower order and less uniform hydration of the maltodextrin chains, correlating with a reduced proportion of short maltooligosaccharides, such as DP2 to DP6, and the presence of DP8, a very high weight-average molecular weight, and a high Pd (9.6), which contributed to significantly lower solubility and water absorption of this maltodextrin. Maltodextrins derived from the starch of colored potato varieties possess excellent functional properties, including nearly 100% solubility and enhanced water absorption capacity due to their fine microstructure, which facilitates hydration and the diffusion of water into the interior of the molecules. It has been confirmed that the technological properties of maltodextrins are primarily influenced by DP and the resulting number-average and weight-average molecular weights, as well as Pd, rather than DE.

It was further observed that maltodextrins derived from the starch of yellow potato varieties (MT and ML) form soft gels, whereas those from colored potatoes form hard gels. This difference is attributed to variations in the composition of maltooligosaccharides, the content of non-carbohydrate components—particularly fat—and differences in the molecular weights of these maltodextrins. Notably, maltodextrins derived from the starch of colored potatoes exhibit significantly higher solubility and water-binding capacity compared to those from light-colored potato varieties, particularly Tajfun. Additionally, they possess a lower SPAN value (2.0), indicating a more uniform distribution, which is advantageous in the food industry. The objective was to optimize this parameter to ensure consistent texture, flavor, and product stability. A significantly higher SPAN value results in instability in various systems, such as during powder segregation or rapid emulsion separation (as observed in the MT and ML samples). It can be inferred that maltodextrins obtained from the starch of red and purple potatoes are suitable for use in the food industry due to their excellent functional properties, including low SPAN, complete solubility, and good water-binding capacity. Nonetheless, further research employing a phenomenological methodology is necessary to analyze the mechanical state of the structure of biomaterials, such as maltodextrins obtained from the starch of potatoes with different flesh colors, considering the physicochemical properties already examined in this study. This may serve as a foundation for further investigation into the behavior of these materials, with the aim of optimizing their application and regulating the production processes involving them.

## Figures and Tables

**Table 1:** DE of investigated maltodextrin.

Maltodextrins from Isolated Starch from Potatoes		DE, %
The color of potato flesh	Variety of potatoes	
Yellow	Tajfun (MT)	18 ± 0.0 ^d^*
	Lord (ML)	16 ± 0.3 ^b^
Purple	Blue Star (MBS)	17 ± 0.1 ^c^
	Violetta (MV)	18 ± 0.0 ^d^
Red	Magenta Love (MML)	15 ± 0.2 ^a^

* Presented data are mean values ± standard deviation (values signed with the same letters); in particular, columns are not significant at 0.05 level of confidence (*n* = 2).

**Table 2:** Maltodextrin Particle Size Distribution and polydispersity index (PDI).

Maltodextrins from Isolated Starch with Potatoes		≤30	30–70	>70	D10	D50	D90	PDI (SPAN)
The Color of Potato Flesh	Variety of Potatoes							
Yellow	Tajfun	75.55 ± 5.91 ^a^*	23.69 ± 5.00 ^d^	0.76 ± 0.88 ^b^	2.44 ± 0.29 ^a^	14.86 ± 2.59 ^bc^	44.78 ± 6.44 ^c^	2.86 ± 0.08 ^c^
	Lord	90.84 ± 1.77 ^c^	9.16 ± 1.77 ^b^	ND **	3.29 ± 0.17 ^b^	11.63 ± 0.65 ^ab^	29.49 ± 1.69 ^ab^	2.25 ± 0.01 ^bc^
Purple	Blue Star	80.84 ± 3.81 ^b^	19.09 ± 3.74 ^c^	0.06 ± 0.00 ^a^	4.74 ± 0.36 ^c^	16.52 ± 1.29 ^c^	37.72 ± 3.17 ^bc^	2.00 ± 0.01 ^a^
	Violetta	95.98 ± 0.75 ^d^	4.02 ± 0.75 ^a^	ND	3.17 ± 0.06 ^b^	10.54 ± 0.24 ^a^	24.55 ± 0.77 ^a^	2.03 ± 0.02 ^a^
Red	Magenta Love	96.69 ± 0.56 ^d^	3.31 ± 0.56 ^a^	ND	3.19 ± 0.06 ^b^	10.27 ± 0.21 ^a^	23.74 ± 0.67 ^a^	2.00 ± 0.02 ^a^

* Presented data are mean values ± standard deviation (values signed the same letters); in particular, columns are not significant at 0.05 level of confidence (*n* = 3). ** ND—not detected.

**Table 3:** Non-carbohydrate components of maltodextrin.

Maltodextrins from Isolated Starch with Potatoes		Protein (%) 6.25	Fat (%)	Ash (%)	Phosphorus (%)
The Color of Potato Flesh	Variety of Potatoes				
Yellow	Tajfun	0.22 ± 0.01 ^b^*	0.43 ± 0.01 ^b^	0.30 ± 0.01 ^c^	0.056 ± 0.00 ^c^
	Lord	0.19 ± 0.00 ^a^	0.47 ± 0.01 ^c^	0.20 ± 0.01 ^a^	0.036 ± 0.00 ^a^
Purple	Blue Star	0.23 ± 0.01 ^b^	0.51 ± 0.00 ^d^	0.35 ± 0.01 ^d^	0.071 ± 0.00 ^e^
	Violetta	0.23 ± 0.00 ^b^	0.37 ± 0.01 ^a^	0.30 ± 0.01 ^c^	0.064 ± 0.00 ^d^
Red	Magenta Love	0.23 ± 0.00 ^b^	0.43 ± 0.01 ^b^	0.26 ± 0.00 ^b^	0.044 ± 0.00 ^b^

* Presented data are mean values ± standard deviation (values signed the same letters) in particular columns are not significant at 0.05 level of confidence (*n* = 2).

**Table 4:** The maltooligosaccharide profile in the maltodextrins.

The Color of Potato Flesh	Variety of Potatoes				
	Yellow		Purple		Red
The Kind of Maltooligosaccharide (g/100 g d.m.)	MT Tajfun	ML Lord	MBS Blue Star	MV Violetta	MML Magenta Love
glucose	0.32 ± 0.02 ^a^*	1.50 ± 0.04 ^c^	1.62 ± 0.11 ^c^	1.36 ± 0.10 ^b^	1.64 ± 0.12 ^c^
maltose	2.30 ± 0.05 ^a^	8.51 ± 0.15 ^b^	9.73 ± 0.60 ^c^	8.43 ± 0.54 ^b^	10.18 ± 0.61 ^c^
maltotriose	4.57 ± 0.07 ^e^	11.87 ± 0.23 ^b^	12.03 ± 0.80 ^b^	11.24 ± 0.70 ^b^	12.80 ± 0.85 ^b^
maltotetraose	4.57 ± 0.03 ^a^	7.93 ± 0.14 ^b^	8.11 ± 0.52 ^c^	7.45 ± 0.48 ^b^	8.55 ± 0.57 ^c^
maltopentaose	2.99 ± 0.06 ^a^	11.92 ± 0.23 ^b^	13.53 ± 0.97 ^c^	11.82 ± 0.85 ^b^	14.15 ± 1.01 ^c^
maltohexaose	6.44 ± 0.12 ^a^	22.11 ± 0.35 ^c^	13.68 ± 1.11 ^b^	12.13 ± 0.98 ^b^	12.53 ± 0.97 ^b^
maltoheptaose	7.14 ± 0.12 ^e^	6.60 ± 0.10 ^d^	2.87 ± 0.20 ^b^	3.33 ± 0.27 ^c^	2.16 ± 0.12 ^a^
maltooktaose	4.35 ± 0.03	ND **	ND	ND	ND
SUM	32.68 ± 0.60	70.44 ± 0.99	61.57 ± 4.11	55.76 ± 3.98	62.01 ± 4.21

* Presented data are mean values ± standard deviation (values signed the same letters) in particular rows are not significant at 0.05 level of confidence (*n* = 3), ** ND—not detected.

**Table 5:** The number-average (Mn) and weight-average (Mw) molecular weights and particle size distribution coefficients (Pd) of the analysed maltodextrins.

Maltodextrins from Isolated Starch with Potatoes	Mn	Mw	Pd [-]
Variety/The Color of Potato Flesh	·10^−3^ [g/mol]		
Tajfun (yellow)	2.55	24.35	9.6
Lord (yellow)	2.97	4.27	1.4
Blue Star (purple)	2.63	4.13	1.6
Violetta (purple)	4.57	5.89	1.3
Magenta Love (red)	2.00	2.99	1.5

**Table 6:** Water-binding capacity (WBC) and solubility (S) of maltodextrins according to the Leach method [35].

Maltodextrins from Potato Starch		WBC 25 °C	WBC 40 °C	Solubility(S) 25 °C	Solubility (S) 40 °C
The Color of Potato Flesh	Variety of Potatoes				
		(g water/g dm)		(%)	
Yellow	Tajfun	4.97 ± 0.96 ^b^*	ND **	76.45 ± 4.53 ^a^	86.56 ± 1.27 ^a^
	Lord	13.15 ± 4.04 ^c^	ND	100.00 ± 1.21 ^b^	99.97 ± 0.37 ^b^
Purple	Blue Star	2.40 ± 0.64 ^a^	ND	100.00 ± 4.73 ^b^	97.82 ± 1.96 ^b^
	Violetta	11.63 ± 1.50 ^c^	ND	100.00 ± 0.68 ^b^	100.15 ± 0.97 ^b^
Red	Magenta Love	22.46 ± 0.66 ^d^	ND	100.00 ± 0.08 ^b^	99.22 ± 0.54 ^b^

* Presented data are mean values ± standard deviation (values signed the same letters) in particular columns are not significant at 0.05 level of confidence (*n* = 2). ** ND—not detected.

**Table 7:** Water-binding capacity (WBC) and solubility (S) of maltodextrins according to the method described by Li et al. [6].

Maltodextrins from Potato Starch		WBC 25 °C	WBC 40 °C	Solubility (S) 25 °C	Solubility (S) 40 °C
The Color of Potato Flesh	Variety of Potatoes				
		(g water/g dm)		(%)	
Yellow	Tajfun	4.30 ± 0.00 ^a^*	16.62 ± 0.12 ^a^	73.15 ± 0.07 ^a^	85.38 ± 0.11 ^a^
	Lord	32.98 ± 2.83 ^c^	48.53 ± 13.09 ^c^	98.33 ± 1.63 ^b^	98.20 ± 0.47 ^b^
Purple	Blue Star	5.32 ± 0.19 ^b^	69.73 ± 9.99 ^d^	97.34 ± 0.03 ^b^	97.91 ± 0.20 ^b^
	Violetta	ND **	ND	100.00 ± 1.46 ^c^	100.00 ± 3.15 ^c^
Red	Magenta Love	ND	29.52 ± 1.48 ^b^	100.00 ± 0.80 ^c^	97.16 ± 0.64 ^b^

* Presented data are mean values ± standard deviation (values signed the same letters); in particular, columns are not significant at 0.05 level of confidence (*n* = 2). ** ND—not detected.

**Table 8:** Rheological parameters of potato maltodextrin samples.

Rheological Parameters		Tajfun (MT)	Lord (ML)	Blue Star (MBS)	Violetta (MV)	Magenta Love (MML)
**G_e_**	[Pa]	0.0194 ± 0.000 ^a^*	0.0434 ± 0.001 ^b^	1.790 ± 0.008 ^d^	7.480 ± 0.173 ^e^	1.590 ± 0.007 ^c^
**J_e_**	[1/Pa]	51.546 ± 0.448 ^e^	23.041 ± 0.542 ^d^	0.559 ± 0.016 ^b^	0.134 ± 0.003 ^a^	0.629 ± 0.014 ^c^
**G_N_^0^**	[Pa]	0.560 ± 0.017 ^c^	0.551 ± 0.016 ^b^	4.886 ± 0.076 ^a^	60.686 ± 0.684 ^e^	4.499 ± 0.073 ^d^
**J_N_^0^**	[1/Pa]	1.786 ± 0.008 ^d^	1.815 ± 0.008 ^e^	0.205 ± 0.003 ^b^	0.016 ± 0.002 ^a^	0.222 ± 0.004 ^c^
**η_0_**	[Pas]	0.875 ± 0.006 ^a^	1.030 ± 0.005 ^b^	72.000 ± 1.775 ^d^	359.000 ± 3.605 ^e^	32.600 ± 0.947 ^c^
**k**	[-]	27.866 ± 0.569 ^e^	11.696 ± 0.316 ^d^	1.730 ± 0.141 ^a^	7.113 ± 0.0.374 ^c^	1.830 ± 0.143 ^b^
**τ** **_m_**	[s]	45.103 ± 0.693 ^d^	23.733 ± 0.613 ^b^	40.223 ± 0.856 ^c^	47.995 ± 1.015 ^e^	20.503 ± 0.801 ^a^
**τ** **_0_**	[s]	1.563 ± 0.007 ^a^	1.869 ± 0.009 ^b^	14.736 ± 0.960 ^e^	5.916 ± 0.089 ^c^	7.246 ± 0.152 ^d^
**ω** **_0_**	[rad/s]	0.640 ± 0.016 ^e^	0.535 ± 0.014 ^d^	0.068 ± 0.002 ^a^	0.169 ± 0.004 ^c^	0.138 ± 0.003 ^b^
**L**	[-]	28.866 ± 0.386 ^e^	12.696 ± 0.384 ^d^	2.730 ± 0.076 ^a^	8.113 ± 0.186 ^c^	2.830 ± 0.078 ^b^
**M_e_**	[kg/mol]	(4.80 ± 0.14) × 10^6^	(4.87 ± 0.14) × 10^6^	(5.50 ± 0.14) × 10^5^	(4.42 ± 0.12) × 10^4^	(5.97 ± 0.16) × 10^5^
**M_chem_**	[kg/mol]	(1.38 ± 0.06) × 10^8^	(6.19 ± 0.16) × 10^7^	(1.50 ± 0.07) × 10^6^	(3.59 ± 0.09) × 10^5^	(1.69 ± 0.08) × 10^6^
**ζ**	[nm]	199.663 ± 2.515	200.744 ± 1.568	96.986 ± 1.592	41.879 ± 0.712	99.691 ± 1.368

* Presented data are mean values ± standard deviation (values signed the same letters); in particular, rows are not significant at 0.05 level of confidence (*n* = 3).

## Data Availability

Data are contained within the article.

## Supplementary Materials

The following supporting information can be downloaded at: https://www.mdpi.com/article/10.3390/molecules31122121/s1, Table S1: Pearson’s correlation.
